# Quantification of Exposure to Risk Postures in Truck Assembly Operators: Neck, Back, Arms and Wrists

**DOI:** 10.3390/ijerph17176062

**Published:** 2020-08-20

**Authors:** Mohsen Zare, Julie Bodin, Jean-Claude Sagot, Yves Roquelaure

**Affiliations:** 1 Univ Angers, CHU Angers, Univ Rennes, Inserm, EHESP, Irset (Institut de Recherche en Santé, Environnement et Travail)—UMR_S 1085, F-49000 Angers, France; julie.bodin@univ-angers.fr (J.B.); yves.roquelaure@univ-angers.fr (Y.R.); 2 ERCOS Group (Pôle), Laboratory of ELLIAD-EA4661, UTBM-University of Bourgogne Franche-Comté, Belfort 90001, France; jean-claude.sagot@utbm.fr

**Keywords:** ergonomics, quantitative measurement, risk evaluation, musculoskeletal disorders, automotive assembly plant

## Abstract

The study assessed the proportion of time in risky postures for the main joints of the upper limbs in a truck assembly plant and explored the association with musculoskeletal symptoms. Fourteen workstations (13 individuals) of a truck assembly plant were selected, and seven sensors were placed on the body segments of the participants. The sensors included tri-axial accelerometers for the arms and back, inclinometers for the neck and electro-goniometry for quantifying flexion/extension of the right and left hands. The proportions of time in moderate awkward postures were high at all workstations. Neck and wrist excessive awkward postures were observed for most workstations. The average values of the 91st percentile for back flexion and right/left arm elevation were 25°, 62°, and 57°, respectively. The 91st and 9th percentile averages for neck flexion/extension were 35.9° and −4.7°, respectively. An insignificant relationship was found between the percentage of time spent in awkward upper limb posture and musculoskeletal symptoms. The findings provide objective and quantitative data about time exposure, variability, and potential risk factors in the real workplace. Quantitative measurements in the field provide objective data of the body postures and movements of tasks that can be helpful in the musculoskeletal disorders (MSDs) prevention program.

## 1. Introduction

Musculoskeletal disorders (MSDs) are the main cause of occupational disease in France and they represented more than 80% of all diagnosed occupational diseases in recent years [1]. Roquelaure et al., (2006), in a cohort study, reported a high prevalence of MSD symptoms for cyclical (under time pressure) and repetitive tasks. For example, the prevalence rate of at least one MSD symptom among males in the automotive and transportation sectors was 20% higher than in other occupational sectors [2]. Work-related MSDs currently pose a challenge for many automotive industries due to exposure to ergonomic risk factors (such as physical risks). This industry involves tasks with many risk factors such as awkward postures and movements, and hand-intensive tasks [3]. The car industry needs strategies and approaches to control risk exposure and to reduce work-related MSDs. Most of these industries have developed ergonomic programs, which focus on physical ergonomics [3,4].

Assessing physical risk exposure is mostly qualitative in the ergonomic program of automotive industries. Qualitative/semi-qualitative methods (observational checklist) are useful as screening tools for identifying critical MSDs risk factors, but quantitative measurements might provide reliable information to create new improvement strategies. Current observational assessment methods used in the automotive industry reveal little evidence of exposure dose for awkward body/limb postures. They are unable to show variability of exposure within and between operators because of measuring one operator (mainly experienced one) for a workstation [5]. Furthermore, the precise risk exposure time and the degree of flexion/extension, particularly for micro postures, are not often measurable by qualitative/semi-qualitative methods [6]. These methods often ignore the duration of exposure to extended moderate risks in one task when a slight proportion of high-risk exposure is identified. Recent studies have shown that exposure to extended moderate risk factors might have a synergistic effect on exposure to risks [7].

This study focused on the quantitative measurement of movements and postures of truck assembly operators who are susceptible to the development of musculoskeletal symptoms, particularly in the upper limbs and back. Several studies have focused on quantitative measurement of awkward postures in various occupational activities due to the development of measurement techniques such as the Inertial Measurement Unit (IMU) in recent years [8,9,10]. Although truck assembly operators are exposed to a range of risk factors in different situations, few studies have quantified the exposure of body segments to awkward postures among this occupation [11,12]. Granzow et al. reported awkward postures of trunk and upper arms for forestry workers [12]. McClellan et al. [13] and Punnett et al. [14] have only reported shoulder loading in the automobile assembly plant, quantitatively. Nordander et al. [11,15], in a series of studies with direct measurement methods, establish a dose–response relationship between physical risk factors and hand/elbow/neck/shoulder musculoskeletal disorders. Even though they analyzed various types of work, few data concerning automotive assembly plants were reported.

This study addresses the physical risk factors (awkward posture) among truck assembly operators. This pilot study aimed to assess the proportion of time in an awkward posture for a series of truck assembly workstations and to explore the association between the body segments’ postures and MSDs symptoms.

## 2. Materials and Methods

### 2.1. Workplace Description

This study was conducted in a sector of a truck assembly plant in France. The company divided the working area into three smaller zones [16] named Improvement Groups (IG; Table 1). These zones contained 14 workstations that we broke down into several positions in order to facilitate data collection. However, we tried to record data as close as possible to the typical working day. The characteristics of each IG, the number of workstations and tools, operators, and types of tasks are presented in Table 1.

### 2.2. Study Participants

Thirteen operators participated in our study (IG1 = 4; IG2 = 5 and IG3 = 4 operators). They rotated between workstations in an IG every two hours. Temporary operators without enough experience and operators with a history of musculoskeletal disorders were excluded. All the participants were men, the mean age was 39.0 (±8.7) years, and the mean length of work experience in the current job was 13.9 (±7.3) years. The mean weight and height of the participants were 72.8 Kg (±8.8) and 175.5 cm (±5.9), respectively. All subjects consented to participate in the investigation, and the written informed consent was obtained, individually. The cycle time for each workstation was 8 min. The operators who rotationally worked at different workstations of an IG were recorded for all the workstations. The data recording was performed in the morning or afternoon at the start of a shift, and 126 cycles were recorded for the 13 operators who worked at 14 workstations (each workstation recorded at least four cycles).

This study is part of a larger study that was approved by two concerned national committees: 1. The Comité consultatif sur le traitement de l’information en matière de recherché dans le domaine de la santé (CCTIRS n°01-215). 2. The Commission nationale de l’informatique et des libertés (CNIL n°901 273).

### 2.3. Measurement

**Figure 1:**
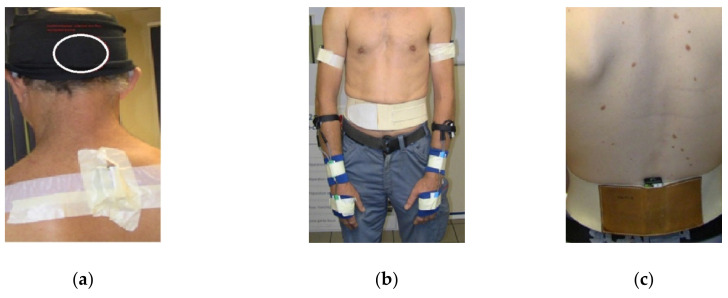
Placement of the sensors on the different body segments: (**a**) two inclinometers placed on the occipital bone and on the cervicothoracic spine at C7-T1 to measure the flexion/extension of the neck; (**b**) two tri-axial accelerometers placed in the lateral side of the right/left arms; (**c**) one accelerometer placed at L3 on the lower back.

Seven sensors, including three triaxial accelerometers and two inclinometers (TEA, Nancy, France), were used to collect data. The sensors were placed on the participants (Figure 1) based on the methods already used in the previous studies [17,18].

Two triaxial accelerometers were placed on the lateral side of the right/left arms in the middle of the humerus. The third accelerometer was placed on the third lumbar spine vertebra (L3). All accelerometers were placed with the vertical y axis. Three signals at orthogonal axes of the sensor represented the acceleration of earth gravity between +1 g and −1 g, sampled at a frequency of 128 Hz and the resolution of 3 mg.

To measure the flexion/extension of the neck, we placed two inclinometer sensors on the occipital bone (a saucer-shaped membrane bone situated on the lower part of the cranium) and on the cervicothoracic spine: last cervical vertebra and first thoracic vertebra (C7-T1) [19]. The accuracy of the inclinometer was 1° if <15° and 2° if >15°. Its resolution and frequency were 12 bits and 16 Hz, respectively.

Two goniometric sensors (Biometrics Ltd., Newport, UK) were fixed over the third metacarpal bone of the hand and to the distal dorsal side of the forearm to measure flexion/extension of the right and left hands. The accuracy and frequency of measurement by goniometer were 2° and 32 Hz, respectively.

The data acquired from the three types of sensors registered in the data logger (attached to the operator’s belt) that was configured with the sensors wirelessly. Then, the registered data was transferred to the CAPTIV L7000 version 2.3.10 (TEA, Nancy, France). This software displays each measurement signal of a body segment over time, separately. The data were also synchronized with the videos in the software.

### 2.4. Reference Position

The reference position, anatomically, is described as the human body upright, feet close together, the arms beside the body, and the palms facing inward [17]. All sensors were regulated to zero when the subjects took this reference position at the start of measurement. However, recent literature cites the importance of considering the morphological and functional differences between individuals [19]. The reference position of each operator was therefore re-recorded at the beginning and the end of data recording for each workstation, while the operator maintained his relaxed position for about 5 s. The mean over 5 s measurement was used as a reference position to calculate the angles of movements of body parts.

### 2.5. Data Processing

Data processing was carried out in Scilab version 5.5.2 (open-source alternatives to Matlab; Enterprises Scilab, Paris, France). The primary aim of the data processing was to obtain the right/left arm and back posture from the acceleration signal, the angle between head and upper back, i.e., flexion/extension of the neck, and flexion/extension of the wrist from zero position (corresponding to the wrist posture in alignment with the forearm). The inclinometer and goniometric sensors provided angular data, which required no special pretreatment, except filtering of signals (to eliminate vibrations and micro-movements).

However, the accelerometer data required various data processing, such as calculation of the angles and signal filtering. The accelerometer measures the magnitude (*ρ*), inclination (*φ*) and direction (*θ*) of the body segment acceleration. The spherical coordinates (*ρ, φ, θ*) describe the position of the sensor. However, each sensor comprises three uniaxial accelerometers that were mounted orthogonally according to x axis, y axis, and z axis. The initial signals have to be converted from an orthonormal vector of the sensor into the spherical coordinate system, via the change of basis of the vectors:

$$x=\rho \;sin\left(\theta \right)cos\left(\varphi \right);\;y=\rho \;sin\left(\theta \right)\;cos\left(\varphi \right);\;z=\rho \;cos\left(\theta \right)$$

where *ρ* >= 0; 0° ≤ *θ* ≤ 180°; −180° ≤ *φ* ≤ 180°.

During static conditions, *ρ* = gravitation (*ρ* ≈ g ≈ 9.81 ms^−2^), *φ* = the extent of inclination relative to vertical and *θ* = the direction of inclination.

According to the literature, it is assumed that the conditions are quasistatic, or at least that the dynamic acceleration component does not influence the calculation of inclination (*φ*) [17]. The following equation was used to convert Cartesian coordinates into spherical data:

$$\varphi ={tan}^{-1}\left(\frac{y}{\sqrt{x^{2}+z^{2}}}\right)$$

where x, y, z = three axes of accelerometers.

A low-pass Butterworth filter of the fourth order with a cut-off frequency of 5 Hz [20] was used to distinguish periods of activity/rest of a body segment.

### 2.6. Classification of Risk Exposure

Angle measurements were classified into several categories based on predetermined thresholds (the thresholds of the in-house observational method [21] used in the factory). The risk assessment procedure was to compare the angle measured in a joint with these threshold limits (Table 2). The thresholds were similar to those reported in the previous studies [22,23,24,25], and in ISO 11226: 2000 [26]. Low, moderate and high-risk factors were indicated as green, yellow and red, respectively, in the factory. If a static awkward posture for a body segment lasted for at least 5 s in cycle time (8 min), it was considered as a risk factor [26]. The percentage of the time was calculated for the angles of the body segments that fell in the defined risk zone, the 9th and 91st percentiles.

### 2.7. Self-Reported Musculoskeletal Symptoms

The subjects determined the pain or discomfort for each body region (the neck, arms/shoulders, elbows/forearms, hands/wrists, and back) at the moment of filling out the questionnaire in a scale from 0 = *no pain/discomfort* to 10 = *intolerable pain/discomfort* [27]. Two operators did not return the questionnaire, and they were excluded from statistical analysis.

### 2.8. Statistical Analysis

We used descriptive statistics (such as percentage of time in awkward posture, percentile, mean and standard deviation of angles) to present the proportion of risk exposure in a cycle time at different workstations. Furthermore, the Spearman correlation was used to explore the relationship between musculoskeletal symptoms with the percentage of risk exposure time. *p*-value less than 0.05 indicate statistical significance, and SPSS version 24.0 (IBM Statistics, Chicago, IL, USA) was used.

## 3. Results

### 3.1. Neck and Back Risk Exposure

The neck was in moderate flexion/extension (yellow) for more than 30% of the cycle time for workstations one to four at IG1. Neck flexion/extension at high-risk levels was almost 3% at different workstations at IG2 (Table 3). At IG3, extreme neck flexion/extension was measured for 14.9% of the cycle time for assembly of the Euro 5 Selective Catalytic Reduction (SCR) tank on the chassis. The operators had to assemble various small parts such as fasteners, retainers, and cable ties on the Euro 5 SCR tank, which required extra attention and focus, and caused extreme neck flexion. However, the proportion of the cycle time on extreme neck flexion/extension posture was 6.5% for the new generation of SCR tank (Table 4).

Table 5 shows the percentage of neck symptoms among the respondents. The Spearman correlation showed that the relationship between the accumulated neck exposure to high and moderate awkward posture with the musculoskeletal symptoms of the neck was not statistically significant (*r* = 0; *p* = 1).

Excessive awkward lower back posture (red) was measured for less than 1% of the cycle time at IG1, except at workstation four (more than 2% of cycle time). However, exposure to moderate awkward posture for the back was considerable at different workstations (Table 3). Maximum exposure of lower back to high and moderate risk levels was 2.8% and 24.6% of cycle time, at IG3, respectively. Lower back awkward posture was almost related to picking assembly parts from the pallet and the assembly of hidden components. The percentage of respondents showing lower back symptoms at the time of answering the questionnaire was 27% (Table 5). The Spearman correlation showed a weak correlation between the percentage of exposure to back awkward posture and lower back MSDs symptoms (*r* = 0.16; *p* = 0.63).

### 3.2. Arms Risk Exposure

The right arm was in the high-risk level for 3.3% of cycle time for workstation one (right boarding step) and 5.2% of cycle time for workstation two (left boarding step; Table 3). The awkward postures of arms in IG1 were related to assembly of the boarding steps—cabling and tightening to the side of the truck and over the boarding step. Exposure to moderate risk was high for all workstations of IG2, varying between 17% and 25% of the cycle time (Table 3). Assembly of the lighting box on the bumper required arm elevation of more than 90° in IG2. The height of the wagon for carrying the bumper, and mounting the lighting box on the top of the bumper required excessive right arm elevation. The right arm exposure to high-risk levels was low at most workstations of IG3 (about 1% of cycle time). However, exposure to moderate risk for the right arm was high. The same pattern of the risk exposure was observed for the left arm (Table 4). The percentage of participants showing MSDs symptoms in the arms and elbows (36%) was higher than the other body segments (Table 5). The Spearman correlation showed no significant relationship between the proportions of time exposure to moderate and high awkward postures and musculoskeletal symptoms for both arms/shoulders (*r* = −0.22; *p* = 0.51).

### 3.3. Wrists Risk Exposure

The proportion of exposure to awkward wrist postures was considerable in most workstations (Table 3 and Table 4). The main reason for wrist flexion/extension was tightening using screwdrivers and performing tasks that required extra force such as connecting hoses and pushing/pulling wagons. We did not find a significant correlation between musculoskeletal symptoms of wrists and the exposure time to awkward wrist postures (*r* = −0.24; *p* = 0.48).

### 3.4. Movement of Body Segments

**Figure 2:**
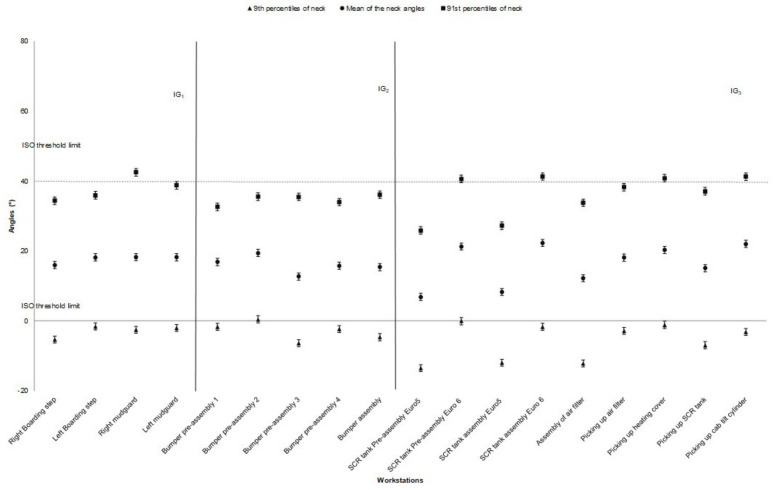
The neck angle (the mean, 91st, and 9th percentiles) for different workstations in the truck assembly plant, (the bars represent standard deviations). The high-risk ISO threshold limit is 40° for neck flexion and 0° for neck extension. Note: IG = Improvement Group; error bars represent standard error.

**Figure 3:**
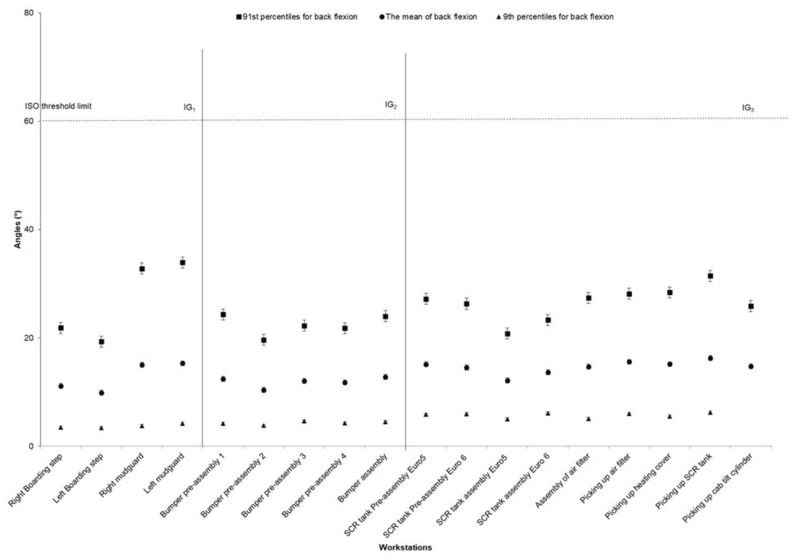
The back angle (the mean, 91st, and 9th percentiles) for different workstations in the truck assembly plant, (the bars represent standard deviations). The high-risk ISO threshold limit is 60° for back flexion. Note: IG = Improvement Group; error bars represent standard error.

The average values of the neck angle for the 91st and 9th percentiles were 35.9° and −4.70°, respectively. Head extension (9th percentiles) was mostly high for preassembly/assembly of SCR tank and air filter assembly. The other workstation showed a similar pattern of head extension (Figure 2). The highest value for back flexion (91st percentile) was for assembly of the mudguards and picking up the SCR. The average back flexion angle for the 91st percentile was 25.4° (Figure 3).

**Figure 4:**
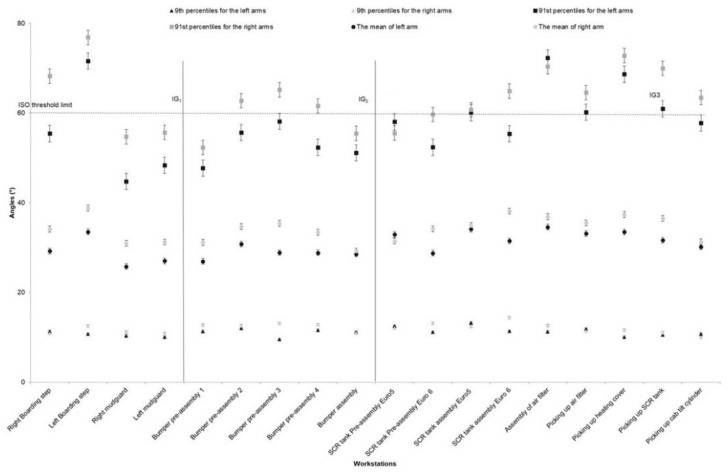
The right and left arms angle values for the mean, 91st and 9th percentiles for different workstations in a truck assembly plant, (the bars represent standard deviations). The high-risk ISO threshold limit is 60° for arm elevation. Note: IG = Improvement Group; error bars represent standard error.

**Figure 5:**
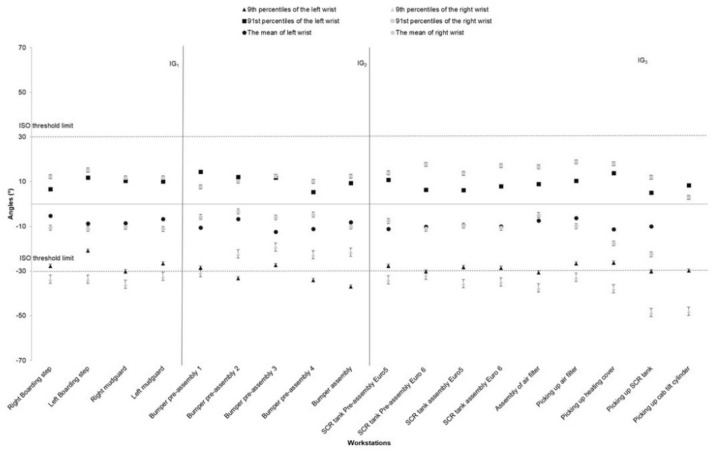
The right/left wrist angles (the mean, 91st, and 9th percentiles) for different workstations in the truck assembly plant, (the bars represent standard deviations). The high-risk ISO threshold limit is ±30° for wrists flexion and extension. Note: IG = Improvement Group; error bars represent standard error.

The highest and the lowest values (91st and 9th percentiles) for the right and left arms elevation are illustrated in Figure 4 for all workstations. The highest angle value for arm elevation (mean approximately 70°) was for the assembly of left and right boarding step systems, preparation of the bumper, picking up the SCR and assembly of the air filter on the truck chassis. Elevation of the right arm was, in general, higher than the left arm, although arm elevation in both arms showed a similar pattern in most of the workstations. We only observed the different patterns between right and left arms elevation in the preassembly and assembly of the SCR tank (Figure 4). Wrist flexion/extension followed the same pattern in various workstations, but the pattern of exposure was different for the left and right wrists. The extension was higher for the left wrist at IG1 and IG3 and lower at IG2. The picking workstation showed the lowest flexion but the higher extension, particularly for the left wrists (Figure 5).

## 4. Discussion

This study was designed to assess the proportion of time exposures to awkward postures for operators in a real assembly setting. We also explored whether the percentage of time exposure to awkward postures is related to MSDs symptoms. The findings showed that exposure to moderate awkward postures was much higher than exposure to high-risk awkward postures; however, exposure to moderately awkward postures increased when the percentage of exposure to high risks was high at one workstation.

**Figure 6:**
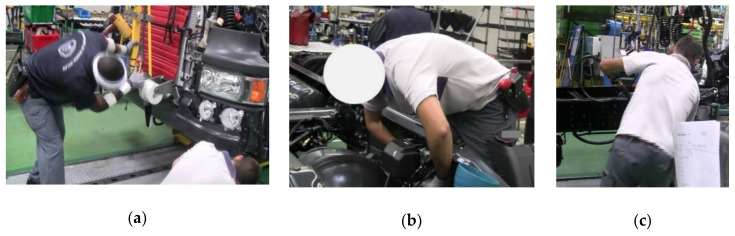
(**a**) Excessive awkward neck and back posture in the assembly bumper workstation due to positioning bumper on the truck’s chassis; (**b**) light cabling on the rear of the mudguard caused awkward back posture; (**c**) tightening with hydraulic screwdrivers required awkward postures and excessive force.

Neck posture was more constrained than other body postures at most workstations. The operators showed less exposure to awkward neck postures for the assembly of new generation SCR tank, thereby showing the design improvement of the new product that reduced risk exposure. More exposure to awkward neck posture was related to positioning the bumper on the truck’s chassis task in the bumper assembly workstation (Figure 6a). Although the exposure time for the neck was high among assembly operators, they did not report high levels of pain at the neck zone. Hansson et al. [28] showed that the neck flexion for the 90th percentile was high in repeated industrial work (approximately 30° for car assembly operators). In another study, the mean of the 90th percentile for neck flexion was between 21° to 42° for the manual and semi-automated lines [29]. We observed similar results in our study (mean neck flexion for the 91st percentile was 35.9°).

The picking up air filter workstation had the highest risk exposure for the back because of picking up/handling the air filter from the pallet. Back flexion was also observed at the mudguard and bumper assembly workstations because of the light cabling on the rear of the mudguard and positioning bumper on the chassis tasks (Figure 6a,b). In this study, back flexion (the mean of 91st percentile for dorsal flexion was 25°) was related to the combination of assembly tasks and picking/handlings. The other studies reported 14° of back flexion for standard light/heavy assembly tasks and 35° for picking up equipment [30]. Burdorf et al. [31] reported that the mean for trunk posture was 12.3 (±8.4) for activities such as welding, pipefitting, repairing, and assembling. Based on work features, task types, and people characteristics, the mean for dorsal flexion would probably be different in various workplaces. We did not find a significant relationship between back pain and awkward posture. Material handling and substantial physical activity, combined with moderate or extreme back flexion might increase the risk of lower back pain [32]. Some studies have shown that the combined effects of leisure time activities, heavy physical workload, and trunk flexion/extension/twisting might cause back pain [33,34].

The percentages of flexion/extension of left/right wrists were high although two risk levels (i.e., minimum risk and high risk) were considered for wrists. Hand/wrist symptoms were relatively high among participants, but the significant correlation was not found between these symptoms and awkward postures. A small sample size might be the main reason for finding no correlation between risk exposure and MSDs symptoms. Balogh et al. [35] reported the mean angles of the right and left wrist flexion/extension to be −16° and −14°, respectively, for standardized assembly tasks in the laboratory, i.e., more than in our study in a real setting. There are usually differences between measurements in real workplaces compared to simulated work tasks in the laboratory. Day-to-day differences in risk exposure are frequent in real workplaces because of variation in work tasks, products (truck models), and individual strategies for performing tasks. These variations are more apparent for micro postures such as wrist and neck flexion/extension. In this study, the right wrist was more exposed to awkward posture than for the left wrist. Similar results were reported in the previous study of various types of work [11,36].

A triaxial accelerometer was used as an inclinometer to measure arm elevation and lower back movement. Several studies have shown that this is a valid method [17,20]. However, this method has limitations because the accelerometer cannot separate rotation from flexion/extension, i.e., back flexion with rotation or abducted/adducted arm cannot be distinguished from arm flexion/extension. We filmed all the workstations measured and synchronized the recordings with the measurements, which enabled us to identify rotation from flexion/extension for the assembly tasks. This method could not quantify the rotation of a body segment.

The most frequent activities analyzed in this study were tightening with electrical/hydraulic pistol grip screwdrivers or angle nutrunner, lifting/handling of parts and assembly of wires, cables, and strips. These tasks required awkward postures and sometimes excessive force (Figure 6c). Although the nature of the truck assembly work was similar at various workstations, we found a considerable difference in exposure to awkward posture between different workstations. The operators had to perform almost identical tasks at the assembly of right and left boarding steps workstations in IG1. Despite the same tasks, the time exposure to risk was different. The reason might be that the operators’ gestures were not the same on the right and left sides. Although we measured right/left workstations on the same day, there were some variations in performing the same tasks between different cycle times. These variations were related to production, deviations, and intraoperator variability. Such variations have been reported as “between-minute” variations, which indicate varied work and might influence the risk of MSDs [37].

Real manufacturing workplaces have limitations that might cause bias in the measurements. The operators might change their strategy to perform a task due to time limitations (performing tasks over-determined cycle times) and/or line stops (because of technical problems) over measurement periods. The Hawthorn effect might also have occurred in our results as the operators’ behaviors were probably influenced due to placing measurement equipment on the body and being filmed while performing the tasks. However, we had several meetings with the operators, the purposes were explained to them, and they volunteered to participate in the experiments. Furthermore, several operators were measured for each workstation, which should have reduced the confounding factors.

Musculoskeletal symptoms measured on a scale of 0 to 10 were dichotomized as absence of pain (score < 5) and presence of pain (score > 5) to measure the correlation between cycle exposure time and musculoskeletal symptoms. Our results have the potential to be biased due to the subjective nature of MSD assessment, which can be strongly influenced by operator moods. It is also possible that some operators were multiskilled and worked in other sectors of the factory, and that the reported musculoskeletal pain might not be truly related to the workstations being measured. Furthermore, this study included a small number of subjects, which may be another reason for a statistically insignificant correlation between awkward postures and MSD symptoms.

## 5. Conclusions

The findings of this study showed that the proportion of time exposure to the excessive awkward posture was high for the neck and wrist among truck assembly operators. They were also exposed to a moderately awkward posture for the neck, back, arms and wrists. MSD symptoms were reported in all body segments, although the correlation between duration of exposure to awkward posture and MSD symptoms was insignificant. The results confirm the variability in exposure between operators and between workstations. Therefore, the results of qualitative/semi-qualitative methods that are measured for experienced operators during a given observation time frame should be interpreted with caution.

Our study showed that exposure to awkward postures was significant and it was necessary to implement an ergonomic program including a reliable evaluation phase and the implementation of solutions with the contribution of the actors, in particular, the operators.

## Figures and Tables

**Table 1:** The characteristics of the Improvement Groups (IG), the number of workstations and tools, operators, and the type of tasks.

Working Group	Number of Workstations	Number of Operators	Number of Operators Participated in the Study	Type of Task	Tool
Improvement group 1 (IG1)	4	6	4	Assembly of left/right boarding steps and left/right mudguards	Electric screwdrivers, hoists, Manual torque wrench
Improvement group 2 (IG2)	5	7	5	Preassembly and assembly of the bumper	Electric and pneumatic screwdrivers, nutrunners, trolleys
Improvement group 3 (IG3)	5	8	4	Preassembly and assembly of Selective Catalytic Reduction (SCR) system, preassembly and assembly of air filter, cab tilt cylinder and material picking	Electric and pneumatic screwdrivers, electric assembly systems

**Table 2:** The threshold limits of flexion/extension for different body segments based on the in-house observational method used in the factory under study (similar to ISO 11226: 2000).

Body Segment	Low Risk (Green)	Moderate Risk (Yellow)	High Risk (Red)
Neck	−30° to 20°	20° to 45°	>45° or <−30°
Back	0° to 20°	20° to 45°	>45°
Arms	0° to 45°	45° to 90°	>90°
Wrists	−30° to 45°	-	<−30° and >45°

**Table 3:** The proportion of time in awkward posture (flexion/extension) for workstations (WS) over one cycle time (Eight min) for Improvement Groups 1 (IG1) and 2 (IG2).

Body Segment	Risk Zone	IG1				IG2				
		WS1 * Right Boarding Steps	WS 2 Left Boarding Steps	WS 3 Right Mudguard	WS 4 Left Mudguard	WS 1 Bumper Pre-Assembly	WS 2 Bumper Pre-Assembly	WS 3 Bumper Pre-Assembly	WS 4 Bumper Pre-Assembly	WS 5 Bumper Assembly
Neck (%)	Low	53.3	68.6	57.1	55.3	58.1	47.1	69.9	59.9	59.1
	Moderate	43.8	29.2	35.1	38.5	40.2	49.8	28.1	37.8	37.1
	High	2.9	2.2	7.8	6.2	1.7	3.1	2.0	2.3	3.8
Lower Back (%)	Low	87.9	93.9	74.1	74.7	81.7	90.6	83.5	84.4	85.0
	Moderate	11.6	6.0	25.0	23.2	17.9	9.0	16.3	14.7	13.0
	High	0.5	0.1	0.9	2.1	0.4	0.4	0.2	0.9	2.0
Right Arm (%)	Low	73.3	68.3	79.8	79.9	81.1	73.2	74.3	76.4	82.7
	Moderate	23.4	26.5	19.7	19.7	17.9	26.0	23.1	23.1	17.1
	High	3.3	5.2	0.5	0.4	1.0	0.8	2.6	0.5	0.2
Left Arm (%)	Low	84.7	76.8	89.7	86.1	89.1	81.4	84.1	85.5	85.5
	Moderate	13.4	19.7	10.2	13.8	10.9	18.0	15.1	14.0	13.9
	High	1.9	3.5	0.1	0.1	0	0.6	0.8	0.5	0.6
Right wrist (%)	Low	83.7	84.7	84.9	84.1	88.3	97.3	95.6	96.8	96.1
	High	16.3	15.3	15.1	15.9	11.7	2.7	4.4	3.2	3.9
Left wrist (%)	Low	89.3	95.4	89.2	90.6	89.7	83.2	93.4	83.8	83.9
	High	10.7	4.6	10.8	9.4	10.3	16.8	6.6	16.2	16.1

* Workstation.

**Table 4:** The proportion of time in awkward postures (flexion/extension) for workstations (WS) in an assembly plant over one cycle time (8 min) for Improvement Group three (IG3); four measurements performed for each WS.

Body Segment	Risk Zone	WS1 * SCR Tank Pre-Assembly		WS 2 SCR Tank Assembly		WS 3 Assembly of Air Filter	WS 4 Picking up Air Filter		WS5 Picking up SCR Tank	
		Euro5 **	Euro6 **	Euro5	Euro6		Air Filter	Heating Cover	SCR Tank	Cab Tilt Cylinder
Neck (%)	Low	66.3	38.5	49.1	46.5	65.6	56.7	62.9	39.4	48.0
	Moderate	29.3	57.0	36.0	47.3	30.9	40.1	32.6	54.7	46.3
	High	4.4	4.5	14.9	6.2	3.5	3.2	4.5	5.9	5.7
Lower Back (%)	Low	88.8	84.2	76.8	79.6	79.4	78.0	73.6	78.9	77.3
	Moderate	10.9	15.4	22.0	19.4	18.9	19.2	24.6	20.1	20.9
	High	0.3	0.4	1.2	1.0	1.7	2.8	1.8	1.0	1.8
Right Arm (%)	Low	72.5	67.9	80.5	74.6	69.6	71.9	69.8	79.6	69.4
	Moderate	26.6	31.5	19.2	25.0	27.8	26.7	27.4	19.2	27.0
	High	0.9	0.6	0.3	0.4	2.6	1.4	2.8	1.2	3.6
Left Arm (%)	Low	75.4	77.5	76.9	84.0	74.3	77.9	78.0	81.0	73.3
	Moderate	23.1	21.6	22.4	15.7	23.0	20.5	20.7	17.5	24.0
	High	1.5	0.9	0.7	0.3	2.7	1.6	1.3	1.5	2.7
Right wrist (%)	Low	83.1	81.3	84.5	87.2	83.2	81.8	66.1	57.9	81.9
	High	16.9	18.7	15.5	12.8	16.8	18.2	33.9	42.1	18.1
Left wrist (%)	Low	89.9	90.7	91.6	89.8	89.9	89.8	90.0	86.7	92.6
	High	10.1	9.3	8.4	10.2	10.1	10.2	10.0	13.3	7.4

* Workstation; ** Two type of Selective Catalytic Reduction (SCR) tanks were prepared and assembled at these workstations.

**Table 5:** The percentage of musculoskeletal symptoms at the time of responding to the questionnaire evaluated using a ten-scale questionnaire (*n* = 13; two non-respondents were excluded).

Body Segment	Musculoskeletal Symptom (*n* = 11)	
	n	%
Neck, VAS * ≥ 5	1	9
Arm, VAS ≥ 5	4	36
Forearms, VAS ≥ 5	4	36
Wrist and hands, VAS ≥ 5	3	27
Fingers, VAS ≥ 5	2	18
Upper back, VAS ≥ 5	2	18
Lower back, VAS ≥ 5	3	27
Hip and thigh, VAS ≥ 5	1	9
Knee and leg, VAS ≥ 5	3	27
Ankle/Foot, VAS ≥ 5	2	18

* visual analog scale for pain.
